# Cell memory-based therapy

**DOI:** 10.1111/jcmm.12646

**Published:** 2015-08-10

**Authors:** Seyed Hadi Anjamrooz

**Affiliations:** Cellular and Molecular Research Center, Kurdistan University of Medical SciencesSanandaj, Iran

**Keywords:** cell memory, cell reprogramming, CMD theory, CMD fluidity, CMD cycle, cell robustness, cell fragility, cell mimicry, entropy, stem cells

## Abstract

Current cell therapies, despite all of the progress in this field, still faces major ethical, technical and regulatory hurdles. Because these issues possibly stem from the current, restricted, stereotypical view of cell ultrastructure and function, we must think radically about the nature of the cell. In this regard, the author's theory of the cell memory disc offers ‘memory-based therapy’, which, with the help of immune system rejuvenation, nervous system control and microparticle-based biodrugs, may have substantial therapeutic potential. In addition to its potential value in the study and prevention of premature cell aging, age-related diseases and cell death, memory therapy may improve the treatment of diseases that are currently limited by genetic disorders, risk of tumour formation and the availability and immunocompatibility of tissue transplants.

## Introduction

**Figure 1:**
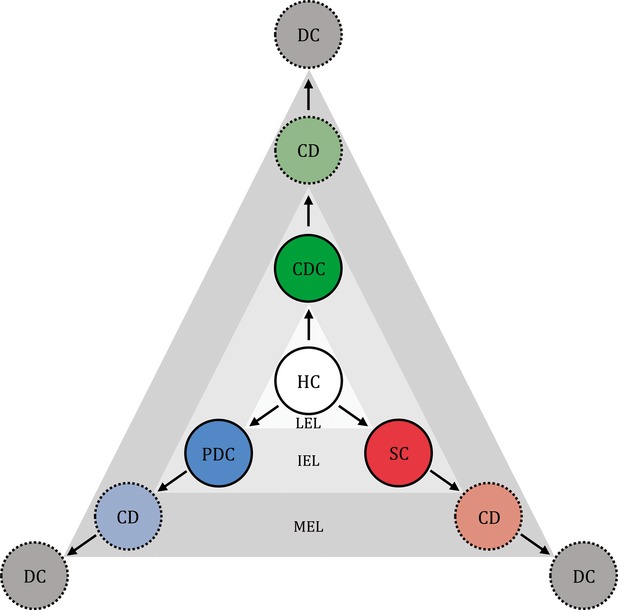
Schematic diagram depicting increases in cell entropy based on the theories of the cellular universe and the holographic triad. Hypothetically, the CMDs of healthy cells will shift to that of damaged cells, dying cells and dead cells with the natural progress toward higher entropy levels. The speed of the cellular entropy increment will be fast, moderate, or slow when the healthy cells shift to CDCs, PDCs or SCs, respectively. HC: healthy cell; CDC: completely diseased cell; PDC: partially diseased cell; SC: senescent cell; CD: cell in the process of dying; DC: dead cell; LEL: low entropy level; IEL: intermediate entropy level; MEL: maximum entropy level.

The cell memory disc (CMD) 1, as a holistic picture of the cell, presents a dynamic, multi-layered system of holographic information storage. The CMD develops gradually over a cell's lifetime and determines all cellular behaviours. Cryptographically speaking, in agreement with the author's theories of the cellular universe and the holographic triad of physical reality 2,3, a ‘trinity’ of master regulators – Oct4, Sox2 and Nanog – coordinately governs pluripotency 4,5 and thereby maintains the blank CMDs of pluripotent stem cells in a state of low entropy (Table1). Normal cellular development and aging as well as cellular stresses help the cells to increase their entropy levels. As the healthy cells proceed towards higher entropy levels, their CMDs will convert to the CMD triad of damaged, dying and dead cells. Damaged cells themselves, based on their speed toward maximum entropy, have a CMD triad consisting of the CMDs of completely diseased, partially diseased and senescent cells (Fig.1).

**Table 1:** Glossary

Entropy: the number of different, indistinguishable microscopic states of a system.
Magnetic resonance spectroscopy (MRS): a non-invasive, diagnostic method that enables the identification and quantification of biochemical changes *in vivo*. MR spectra can be obtained from different nuclei, including carbon (^13^C), nitrogen (^15^N), fluorine (^19^F), sodium (^23^Na) and phosphorus (^31^P). The most used nucleus for clinical applications is hydrogen (^1^H) mainly because of its high sensitivity and abundance. The brain is ideally imaged with H-MRS because of its near lack of motion. However, this technique can also be used for tissues of the thorax, abdomen and pelvis if it is combined with motion-reduction techniques. In addition, the sensitivity of this technique for detecting smaller elements must also be increased for applications in memory-based therapy.
Nanoscopy: a variety of techniques to investigate biology at the nanometre and nanomolar levels.
Cell robustness: a cell's tendency to preserve its basal state both morphologically and functionally.
Cell fragility: the inability of a cell to resist aberrant or forced memorization.
Cell mimicry: the ability to mimic the morphology and behaviour of other cells.
Extracellular vesicles: membrane vesicles that act as vehicles for information transfer. They contain microRNAs, mRNAs, long non-coding RNAs, proteins, glycoproteins, lipids, occasionally genomic DNA and other molecules based on the type of cell secreting them.

**Figure 2:**
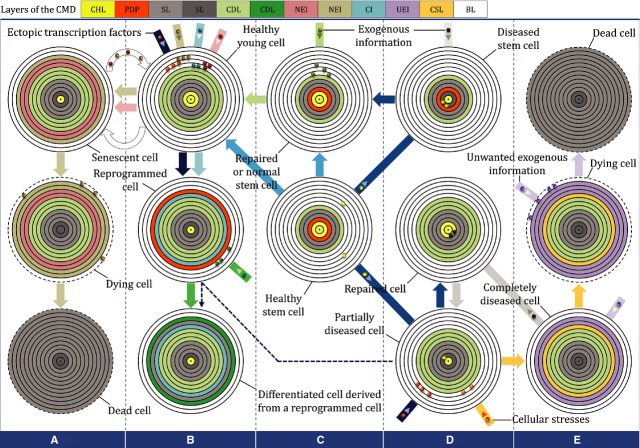
Hypothetical diagram of different cell states based on CMD kinetics. In two regenerative approaches, including cell-based therapy (**B** and **C**) and memory therapy (**C** and **D**), the cells behave in different ways. Each healthy cell can gradually develop completely and then undergo cell death (**A** and **B**). Cells can also be affected by disease-inducing factors from neighbouring diseased cells (black microvesicles) or health-suppressing factors, including cellular stresses and ectopic transcription factors and may thereby become partially diseased (**B** and **D**) or undergo premature cell death (**D** and **E**). Moreover, senescent cells, by transmitting information (microvesicles in the curved arrow), fill up the blank CMD layers of neighbouring young cells and thereby accelerate the transition of young cells towards senescence and death (**A** and **B**). However, healthy stem cells are able to reactivate the silent CHL in the CMDs of neighbouring diseased cells *via* shedding of memory microvesicles (**C** and **D**). CHL: cellular health-related layer; PDP: proliferation and differentiation potential; SL: silent layer; CDL: cell differentiation-related layer; NEI: normal exogenous information; CI: culture-originated information; UEI: unwanted exogenous information; CSL: cellular stress-related layer; BL: blank layer.

Hypothetically, as shown in Figure2, blank layers of the CMD fill up gradually with new information during cell development and aging. Concurrently, in addition to increasing entropy, the cellular health-related layers (CHLs) within the CMD will be inactivated. This natural process of cell senescence has the potential to be echoed in the CMDs of neighbouring cells, including healthy and young cells. Presumably, this is achieved by silencing of the CHLs of neighbouring CMDs that, in turn, speeds up the process by which the blank layers of the CMD are filled with ectopic information and thus instigates premature cell aging. Along this line, previous studies reported that both endogenous and exogenous senescent cells have the potential to shorten the lifespan of their healthy neighbours. For example, in a study conducted by Baker *et al*. (2011), murine senescent cells were selectively cleared, which led to greater resistance against age-related diseases, corrected or delayed tissue dysfunction and extended the health span 6. Furthermore, rodent parabiosis studies have shown that old circulation ages young stem cells 7.

Basically, it is possible that a cell would normally undergo cell death after all of its blank CMD layers have gradually been filled by the entry of controlled information over the cell's lifespan (Fig.2A). However, in the presence of deteriorating cellular stresses, all CHLs may become inactivated, thus allowing the blank layers to fill up quickly with unwanted exogenous information. This is a process by which a cell becomes completely diseased and then undergoes sudden death. In the absence of appropriate stimuli for the reactivation of the silent CHL in the CMD of a partially diseased cell, blank layers of its CMD are inhabited by information about cellular stresses and related unwanted information, which finally, along with the filling of the CMD, leads to premature cell aging and eventually to cell death (Fig.2E). The detrimental effect of cellular stresses on cell aging has been reported previously 8; however, the author's CMD-based outlook may open a new perspective for studies of this topic.

**Figure 3:**
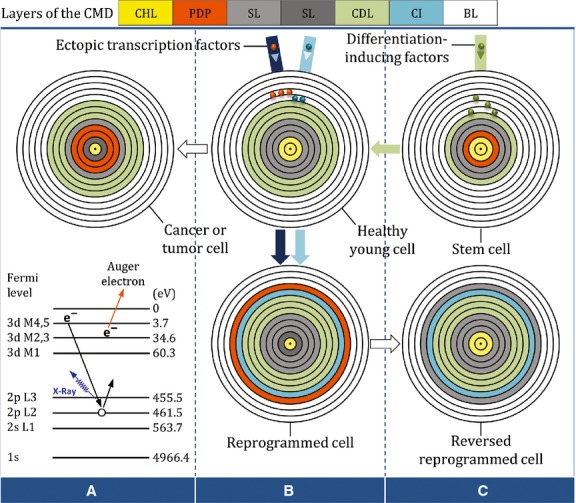
Schematic diagram showing the hypothetical kinetics of CMD layers of proliferation and differentiation potential (PDP). If two silenced layers of the PDP in the CMD of a healthy differentiated cell are reactivated by endogenous X-rays, the cell reacquires pluripotent capacity and becomes tumorigenic or cancerous (**A** and **B**). However, a cell can also be reprogrammed to a pluripotent-like state when a large layer of PDP is generated with the help of exogenous transcription factors (**B**). Virus-based reprogramming, in addition to being the exogenous route to pluripotency, may activate the endogenous path within the host CMD and thereby increase the risk of tumorigenicity. Normally, as illustrated in the CMD of a reversed reprogrammed cell, due to a significant volume of differentiation and culture-originated information, the exogenous pluripotent capacity is temporary and thus will be silenced. Similarly, in the CMD of a multipotent stem cell, the only active layer of PDP will be gradually silenced as cell differentiation proceeds (**B** and **C**). In this regard, in the CMD of an *in vivo* totipotent stem cell, all three layers of PDP are active, while all other layers of the CMD are blank. CHL: cellular health-related layer; SL: silent layer; CDL: cell differentiation-related layer; CI: culture-originated information; BL: blank layer.

Doubtlessly, the interventional control of cell memory *in vivo* is a key issue that should be considered in regenerative medicine. To achieve success with this strategy, a pathological process in the very early stages, prior to any obvious morphological changes, must initially be diagnosed at atomic levels of target tissue voxels with the help of techniques similar to magnetic resonance spectroscopy and nanoscopy (Table1). This is because, along with cell development, aging and cellular stresses, the entry of information into the CMD and its fluidity change the atomic architecture of the CMD. Therefore, researchers and scientists should focus narrowly on the atomic signs of diseases that are formed in the CMD. Based on nuclear physics, it seems possible that the increase in the entropy of the CMD, as a type of energy flow, could fuse some of the cell's atomic nuclei to each other or split them into smaller nuclei. These nuclear reactions, fusion and fission, can generate not only energy but also heavier and/or lighter atoms such as hydrogen (^1^H), carbon (^13^C), nitrogen (^15^N), fluorine (^19^F), sodium (^23^Na) and phosphorus (^31^P). Nuclear fusions and fissions are the natural responses of the atomic structure of the cell by which it attempts to reach a more stable state of equilibrium. Nature only recognizes balance and imbalance; it does not recognize good and evil. Therefore, because greater equilibrium alters the atomic and molecular structure of the cell's normal status, it is not desirable from the biological and physiological perspective. Furthermore, the nuclear-derived energy is a form of information that can fill up some of the CMD's blank layers and thereby negatively affects the cell's lifespan (Fig.2D and E). In addition to the nuclear reactions, electron number and location are also affected by increases in entropy. Based on quantum mechanics, change in electron number or location alters atom shape and behaviour. Such electron reactions may reveal themselves as pathological changes in cell ultrastructure and function. For instance, it is possible that the energy resulting from quantum jumping within the subatomic structure of a differentiated cell initiates a process known as the *Auger effect*, which can generate X-rays. The X-rays produced in this way may reactivate silenced pluripotency layers in the CMD of a host cell and thereby cause the cell to become tumorigenic or cancerous (Fig.3A).

**Figure 4:**
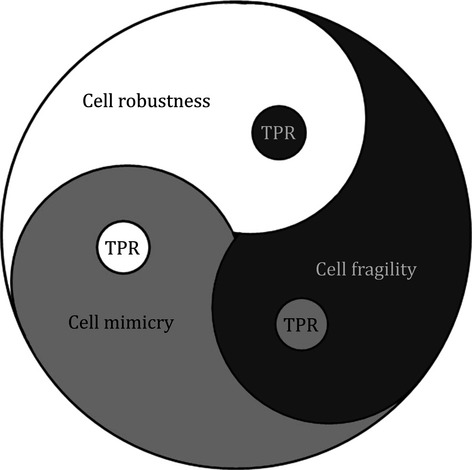
Schematic diagram depicting fundamental cellular behaviours based on the CMD theory. TPR: two-phase region.

In the CMD theory, the three phases of robustness (R-phase) 9–15, fragility (F-phase) 16 and mimicry (Mi-phase) 17–23 constitute the CMD cycle (Table1). At two-phase regions (TPRs), the cell has the capacity to simultaneously exhibit the behaviours of two different phases (Fig.4). Hypothetically, atomic and molecular injuries to the cell could trigger any layer of the CMD. However, the extent of CMD pathology, in addition to injury severity, should depend on the phase of the CMD cycle during which the cellular damage occurs. If injury to the CMD is superimposed on vital layers of cellular health, R-phase cells may become partially diseased, while the F-phase cells could be severely affected or may die. On the other hand, for Mi-phase cells, the extent of pathology would be influenced by the type and activity of the TPR. If damage occurs to other layers without involving CHLs, the CMD cycle may arrest at the Mi-phase. The phenotypes of Mi-phase-arrested cells are likely determined by the type and number of damaged layers. Generally, atomic and molecular changes to the CMD induced by different stressors could manifest in the forms of different diseases.

## CMD restoration

Theoretically, memory therapy would be able to target cells through three complementary approaches: the restoration of the CMD's R-phase in which partially diseased cells are restored to their healthy status, restoration of cells arrested at Mi-phase to their original states, and elimination of irreversible F-phase cells, especially those of the immune system, or blocking of their vesicular release.

For R-phase restoration, the silent CHL within the CMDs of partially diseased cells must be reinvigorated holographically and maintained in an active state. This can be achieved both endogenously and exogenously. The active CHL within the CMDs of partially diseased cells may have a holographic capacity to reactivate the silent CHL upon appropriate stimulation. One possible tool for this purpose is the development of new molecules that can reach and bind to the partially diseased cells and coax them to remember and reactivate their previous normal state. Although this endogenous-based approach seems simple in theory, it may not work in all situations; therefore, we must also consider exogenous options. There is evidence that implicitly supports such a possibility. For example, there are several reported cases of successful treatment of total limbal stem cell deficiency with autologous oral mucosal epithelium 24 or allogeneic stem cells 25. However, it has been widely documented that adult epithelial stem cells maintain their differentiation program, even when grafted onto different body sites. In addition, and more importantly, long-term follow-up demonstrated the presence of autologous limbal stem cells that had not been active in promoting epithelialization before transplantation. A CMD-based explanation for these findings is that the transplantation of undamaged cultured cells, likely in the R-phase of the CMD cycle, reactivated the silent CHL in the CMDs of the diseased cells of the recipient tissue, including those of R-phase stem cells at recessive TPR, *via* the release of effective substances. The repaired cells then restored the previous normal condition of the damaged tissue.

Recently, Snyder and Teng (2012) concluded from previous studies that a substantial amount of spontaneous recovery in the injured spinal cord is not directly related to transplanted cells. This recovery can occur for reasons that are not entirely understood 26. It is possible that much of this recovery is again attributable to the reactivation of silent health-related memory in the CMDs of injured cells, which appears during the resolution of processes such as inflammation, shock, oedema, transient channelopathies and altered perfusion. It has also been reported that bone marrow-derived stromal cells (BMSCs) transfer mitochondria-containing substances to pulmonary alveolar epithelia through gap junction channels. The transferred mitochondria increase the alveolar Adenosine triphosphate (ATP) concentration and likely provide the energy required to reactivate the silent CHL in the CMDs of the injured cells. In this way, BMSCs protect host cells from acute lung injury 27.

These studies took an important further step by uncovering the mysterious role of extracellular vesicles (EVs) in regenerative medicine. Recent findings have noted that EVs (Table1) echo the phenotypes of the cells that produce them and have therapeutic potential 28,29. Therefore, such vesicles may be able to echo the health status of their parent cells. Based on this assumption, the EVs of healthy cells must have the ability to reactivate the silent CHL in the CMDs of neighbouring diseased cells after fusion with the target cell membrane. Similarly, as depicted in Figure2 and considering the results of Baker *et al*. 6, the EVs of damaged cells, including diseased or senescent cells, must also be able to echo the diseased or senescent status of their parent cells, respectively. If this interpretation is correct, then researchers should try to find ways to control the trafficking of these memory vesicles. For example, with respect to the regulatory role of the nervous system in the cell microenvironment 30, governing vesicular trafficking *via* the nervous system could represent an appropriate therapeutic intervention.

In theory, the CMD stores information holographically 1. Based on a *Nature* paper by the Nobel laureate physicist Dennis Gabor 31, each part or subpart of a hologram contains all of the information possessed by the entire hologram 2,3. To simplify, this is similar to seeing the image of an object in a mirror. The image of that object has a non-local property, so that any fragment of the shattered mirror can also show the image of the whole object. Similarly, from a biological perspective, each cell of the human body contains all of the information necessary to generate another whole being. Scientists have successfully used this amazing property to create cloned animals in recent decades. On a smaller scale, it can be assumed that a microvesicle represents a ‘zipped backup’ of all data recorded in the CMD of its cell of origin.

Based on the holographic concept of the CMD, the cell itself is a macrovesicle with a fusion tendency similar to those of microvesicles; perhaps this is why the unexpected cell fate changes that are observed following cell engraftment are consequences of cell fusion rather than transdifferentiation 9,32–37. This CMD-based argument suggests that true transdifferentiation cannot occur in nature. In this line, it has been shown that morphological and molecular data are not sufficient indicators of transdifferentiation 38–41. Although conversions between cells with very similar CMDs, such as α-cells and β-cells or B cells and macrophages, have been proposed to occur 42, they may in fact be the result of the CMD's Mi-phase and not a true and permanent conversion.

Because fused cells have a very low frequency of survival and a risk of aneuploidy, which is a hallmark of cancer cells 43, the development of novel biodrugs similar to the memory microvesicles of healthy cells is a key that could unlock some of the potential of CMD therapy for regenerative medicine. Moreover, owing to uncontrollable exogenous memorizations that occur during the manipulation and culture of patient-derived cells, *in vitro* technologies are not completely reliable tools for studying the molecular mechanisms that truly underlie pathogenesis or for testing potentially useful drugs. Therefore, memory-based biodrugs may offer a good alternative to chemically synthesized drugs with serious side effects. In this regard, as parabiosis experiments indicated that young circulation rejuvenates the stem cell population of elderly mice 7, cell-free blood samples from young people, which contain stem cell-derived vesicles, could be used as a source for biodrug formulation and delivery. Accordingly, the idea of personalized drug therapy brings into focus biodrug formulations from the cord blood of newborns for future medical use. In addition, further development of this biomolecular approach may one day yield effective treatments for some genetic disorders.

Cell memory disc fluidity can manifest in the form of reversible plasticity during Mi-phase, which may be spontaneous 44–46 or intentionally induced if the CMD cycle has been arrested at Mi-phase. For example, during very early passages of hepatocyte reprogramming, newly generated induced pluripotent stem (iPS) cells still retain expression of developmental regulators; this expression could be used to promote differentiation back into the parental cell state 47, suggesting that the iPS state is merely a phenotype of Mi-phase-arrested hepatocytes. In another example, once the memory layer associated with the osteogenic phenotype of Mi-phase-arrested cells of a human bone marrow stromal cell-derived myogenic subclone was silenced, the cells regained their myogenic ability 41. It is thus reasonable to expect that manipulations of the CMD will someday be able to reset Mi-phase-arrested cells to their default states *in vivo*. This strategy may be particularly useful for chronic injuries in which constant activation of healthy stem cells has the potential to result in the accumulation of mutations and the generation of cancer stem cells.

In addition to differentiated cells, the reversible plasticity of stem cells is also a consequence of CMD fluidity. From previous studies 48, we can infer that temporary plasticity is a physiological ability at the Mi-phase of the CMD cycle whereby stem cells preserve their activity and longevity. On this basis, with the development of novel drugs, it will be possible to reverse this process to support tissue regeneration in emergency situations or whenever natural wound-healing signals are not able to restore it. In addition, this strategy may be useful for eliminating camouflaged cancer cells, which are thought to be Mi-phase stem cells masked as non-proliferating cells and hence are resistant to anti-proliferative chemotherapy 49,50.

Cell memory disc restoration of reversible target cells is only one side of the CMD therapy triad; the other is to shorten the lifespan of irreversibly damaged cells of the immune system, including completely diseased cells and senescent cells, by accelerating the process by which the blank layers of their CMDs are filled. One option toward this end is the development of specific stressor drugs similar to those that have been used experimentally to eliminate damaged cells in the mouse 6,51. The rejuvenated immune system then destroys other irreversibly damaged cells whenever they arise. If this system fails, aging and age-related pathologies would develop because irreversibly damaged cells, owing to their relatively full CMDs, are able to accelerate the filling of CMDs of neighbouring healthy and partially diseased cells by delivering information packages in the form of vesicles. In addition to irreversibly damaged cells, a rejuvenated immune system could recognize tumorigenic cells and try to eliminate them.

## CMD therapy *versus* reprogramming therapy

Based on the CMD theory, cell reprogramming is not a new phenomenon but rather an innate dimension of cell behaviour at the Mi-phase of the CMD cycle by which a cell, in an attempt to preserve its viability, temporarily acquires a mimicry phenotype. This process is influenced by the activities of TPRs from the beginning to the end of Mi-phase, so that increases in entropy during F-phase activity in the recessive TPR determines which mimicry phenotype is expressed. However, the mimicry phenotype fades with the increase in R-phase activity in the dominant TPR. Finally, the CMD exits from the dominant TPR, and the cell regains its original phenotype.

In contrast to innate cell reprogramming, intentional reprogramming exerts stress on the CMD. In theory, both reprogramming efficiency and the extent of pathology depend on the phase at which the CMDs are intentionally manipulated. While R-phase cells are mostly resistant to reprogramming and may become partially diseased, F-phase cells show some degree of reprogramming and become severely diseased. It is only Mi-phase cells that may undergo maximum reprogramming both efficiently and durably; however, these Mi-phase-arrested cells and their derivatives later partially or completely show parental-like behaviours depending on the activity level of the hidden R-phase. Therefore, in addition to the low reprogramming rate (Table S1), complete plasticity is a rare event. In this line, it was revealed that reprogrammed cells show persistent expression of genes that were active in their parent cells 23,52–54. Similarly, if reprogrammed cells are derived from diseased cells, the disease status memory of the original cells might be observed in the CMDs of the reprogrammed cells. This layer of disease status even persists in the CMDs of the resulting differentiated cells (Fig.2, dashed arrow). For example, in support of this hypothesis, cardiomyocytes derived by inducing the differentiation of patient-specific iPS cell lines showed disease features similar to those of the patients from whom they were obtained 55.

The F-phase activity at the recessive TPR is necessary for reprogramming Mi-phase cells, but at the same time, it may allow unwanted information to settle in the CMD of intentionally reprogrammed cells. Hence, it is reasonable to observe unwanted cell behaviours. For instance, ‘imprinted’ genes are especially sensitive to environmental signals, and culture-derived signals should not be an exception. Imprinted genes have only a single active copy at any one time. Any culture-derived variation in that copy can be expressed, and there is no backup copy to mask its effects. Thus, again, it is tempting to consider cultured cells, such as reprogrammed cells and their derivatives, as diseased cells, even if the cell only underwent minor manipulations. In this regard, cloned mice derived from uncultured cells with normal imprints showed abnormal expression of imprinted genes 56. In cloned embryos, regardless of the donor cell, the erratic expression of imprinted genes may be responsible for birth defects such as large offspring syndrome 16,17.

Increases in R-phase activity at the dominant TPR may be able to restore Mi-phase-arrested cells to their original state. This CMD-based interpretation is in agreement with those of previous studies that found that the reprogrammed status of a cell is reversible 38,39,44,47. For example, histone modifications, despite their crucial role in genome reprogramming, are reversible 57. However, the reversible capacity of the reprogrammed cells and their derivatives may be incomplete. Cell memory disc kinetics reinforce the hypothesis that exogenous memories created by the reprogramming process in the CMDs of reprogrammed cells can at least partially account for the incomplete reversible capacity of reprogrammed cells toward their previous CMD state. Hence, it seems reasonable to consider ectopic transcription factors as lifespan-shortening factors because they remain as an uninvited layer of silent information in the CMDs of the reversed-reprogrammed cells (Fig.3B and C). If this notion proves to be correct, then the CMDs of iPS-derived cells should also contain this unwanted layer (Fig.2B).

In contrast to cellular reprogramming therapies that actually appear to shift target cells from healthy to diseased states, CMD therapy has the potential to restore partially damaged cells in addition to eliminating completely damaged cells. In tune with previous reports 24,25, while engraftment of uncultured stem cells may be capable of reactivating the silent CHL in the CMDs of diseased cells of the recipient, after transplantation of induced stem-like cells, this layer of the CMD cannot be reactivated. This is because, as supposed above, cultivation and manipulation can make cells sick. Accordingly and in harmony with other studies 17,58–60, it seems likely that only rare R-phase stem cells within the donor cell population, rather than reprogrammed cells, are responsible for reactivating the silent CHL in the CMDs of diseased cells. Unfortunately, these abnormally robust stem cells can also be tumorigenic.

## Conclusion and perspectives

In summary, CMD-based therapy has considerable therapeutic potential and seems to be associated with fewer ethical and safety issues than other cell-based therapies. This hypothetical approach may be highly optimistic; however, many details regarding the molecular, atomic and subatomic kinetics of the CMD during cell damage and repair must be elucidated if we expect this strategy to be applicable in the future. The successful implementation of this type of therapeutic intervention could be an important step forward in regenerative medicine.

## Supporting Information

**Table S1** Low efficiency rates of different cell transformation processes.
