# Supplementary material for: Cell memory-based therapy
Source: J Cell Mol Med. 2015 Aug 10;19(11):2682–9. doi: 10.1111/jcmm.12646 (PMC4627573; doi:10.1111/jcmm.12646)
Supplement: Supplementary file 1 — Table S1 Low efficiency rates of different cell transformation processes. [file jcmm0019-2682-sd1.doc]

**Supporting Information**

**Cell memory-based therapy**

Seyed Hadi Anjamrooz

| **Table S1 Low efficiency rates of** different cell transformation **processes** | | | | |
| --- | --- | --- | --- | --- |
| Original or donor cells | Generated cells or cloned animals | Route | efficiency rate % | Refs |
| Mouse embryonic fibroblasts | Neural progenitors | Dedifferentiation [in vitro] | 0.5–0.7% | Kim J. et al. [*Proc. Natl. Acad. Sci.*](http://www.ncbi.nlm.nih.gov/pubmed?term=Kim J%2C et al.(2011) Direct reprogramming of mouse fibroblasts to neural progenitors. Proc Natl Acad Sci USA 108%3A7838–7843.) *U S A* 2011 |
| Epiblast stem cells | Inner cell mass-like pluripotency | Dedifferentiation [in vitro] | 2% | [Hanna J](http://www.ncbi.nlm.nih.gov/pubmed?term=Hanna J%5BAuthor%5D&cauthor=true&cauthor_uid=23881547). et al. [*Cell Stem Cell*](http://www.ncbi.nlm.nih.gov/pubmed) 2009 |
| Cardiac postnatal or dermal fibroblasts | Cardiomyocyte-like cells | Transdifferentiation [in vitro] | 1.7% | Ieda M. et al. [*Cell*](http://www.ncbi.nlm.nih.gov/pubmed/?term=IedaM%2C+et+al.+(2010)+Direct+reprogramming+of+fibroblasts+into+functional+cardiomyocytes+by+defined+factors.+Cell+142%3A375–386.)2010 |
| Fetal and postnatal fibroblasts | Neurons | Transdifferentiation [in vitro] | 2–4% | [Pang ZP](http://www.ncbi.nlm.nih.gov/pubmed?term=Pang ZP%5BAuthor%5D&cauthor=true&cauthor_uid=21617644). et al. [*Nature*](http://www.ncbi.nlm.nih.gov/pubmed/?term=Pang+ZP%2C+et+al.+(2011)+Induction+of+human+neuronal+cells+by+defined+transcription+factors.+Nature+476(7359)%3A220–223.) 2011 |
| Neurosphere cells | Hematopoietic cells | Transdifferentiation [in vivo] | 0% | [Morshead CM](http://www.ncbi.nlm.nih.gov/pubmed?term=Morshead CM%5BAuthor%5D&cauthor=true&cauthor_uid=11875498). et al. [*Nat. Med.*](http://www.ncbi.nlm.nih.gov/pubmed/?term=Morshead%2C+C.+M.%2C+Benveniste%2C+P.%2C+Iscove%2C+N.+N.+%26+van+der+Kooy%2C+D.+Hematopoietic+competence+is+a+rare+property+of+neural+stem+cells+that+may+depend+on+genetic+and+epigenetic+alterations.+Nat+Med.+8%2C+268-273+(2002).) 2002 |
| Bone marrow stem cells | Donor-derived neurons | Transdetermination [in vivo] | ~0.001% | [Mezey E](http://www.ncbi.nlm.nih.gov/pubmed?term=Mezey E%5BAuthor%5D&cauthor=true&cauthor_uid=12538864). et al. [*Proc. Natl. Acad. Sci.*](http://www.ncbi.nlm.nih.gov/pubmed?term=Kim J%2C et al.(2011) Direct reprogramming of mouse fibroblasts to neural progenitors. Proc Natl Acad Sci USA 108%3A7838–7843.) *U S A* 2003 |
| Bone marrow cells | Donor-derived neurons, astrocytes and microglia | Transdetermination [in vivo] | 1-2% | [Cogle CR](http://www.ncbi.nlm.nih.gov/pubmed?term=Cogle CR%5BAuthor%5D&cauthor=true&cauthor_uid=15121406). et al. [*Lancet*](http://www.ncbi.nlm.nih.gov/pubmed/?term=Cogle%2C+C.+R.%2C+Yachnis%2C+A.+T.%2C+Laywell%2C+E.+D.%2C+Zander%2C+D.+S.%2C+Wingard%2C+J.+R.%2C+Steindler%2C+D.+A.+%26+Scott%2C+E.+W.+(2004)+Lancet+363%2C+1432–1437.) 2004 |
| Human bone marrow and peripheral blood stem cells | Donor-derived endothelial cells in the recipient’s skin and gut | Physiologic differentiation [in vivo] | ~2% | [Jiang S](http://www.ncbi.nlm.nih.gov/pubmed?term=Jiang S%5BAuthor%5D&cauthor=true&cauthor_uid=15548607). et al. [*Proc. Natl. Acad. Sci.*](http://www.ncbi.nlm.nih.gov/pubmed?term=Kim J%2C et al.(2011) Direct reprogramming of mouse fibroblasts to neural progenitors. Proc Natl Acad Sci USA 108%3A7838–7843.) *U S A* 2004 |
| Bone marrow-derived myeloid cells and recipient cardiomyocytes | Bone marrow-derived cardiomyocytes | Cell fusion [in vivo] | ~0.04% | [Fukata M](http://www.ncbi.nlm.nih.gov/pubmed?term=Fukata M%5BAuthor%5D&cauthor=true&cauthor_uid=23989454). et al. [*PLoS One*](http://www.ncbi.nlm.nih.gov/pubmed/?term=Wang+R%2C+Sun+X%2C+Wang+CY%2C+Hu+P%2C+Chu+CY%2C+et+al.+(2012)+Spontaneous+cancerstromal+cell+fusion+as+a+mechanism+of+prostate+cancer+androgen-independent+progression.+PLoS+One+7%3A+e42653.) 2013 |
| Bone marrow cells and recipient cardiomyocytes | Cardiomyocytes | Cell fusion [in vivo] | ~0.75% | [Nygren JM](http://www.ncbi.nlm.nih.gov/pubmed?term=Nygren JM%5BAuthor%5D&cauthor=true&cauthor_uid=15107841). et al. [*Nat. Med.*](http://www.ncbi.nlm.nih.gov/pubmed/?term=Nygren+JM%2C+Jovinge+S%2C+Breitbach+M%2C+Sa¨we´n+P%2C+Ro¨ll+W%2C+et+al.+(2004)+Bone+marrow-derived+hematopoietic+cells+generate+cardiomyocytes+at+a+low+frequency+through+cell+fusion%2C+but+not+transdifferentiation.+Nat+Med+10%3A+494–501.) 2004 |
| Mesenchymal stem cells (MSCs) and small airway epithelial cells (SAECs) | SAECs | Cell fusion [ex vivo] | ~1% | [Spees JL](http://www.ncbi.nlm.nih.gov/pubmed?term=Spees JL%5BAuthor%5D&cauthor=true&cauthor_uid=12606728). et al. [*Proc. Natl. Acad. Sci.*](http://www.ncbi.nlm.nih.gov/pubmed?term=Kim J%2C et al.(2011) Direct reprogramming of mouse fibroblasts to neural progenitors. Proc Natl Acad Sci USA 108%3A7838–7843.) *U S A* 2003 |
| Bone marrow cells and recipient liver cells, cardiomyocytes and Purkinje neurons | Multinucleated cells | Cell fusion [in vivo] | 1%< | [Alvarez-Dolado M](http://www.ncbi.nlm.nih.gov/pubmed?term=Alvarez-Dolado M%5BAuthor%5D&cauthor=true&cauthor_uid=14555960). et al. [*Nature*](http://www.ncbi.nlm.nih.gov/pubmed/?term=Alvarez-Dolado%2C+M.+et+al.+Fusion+of+bone-marrow-derived+cells+with+Purkinje+neurons%2C+cardiomyocytes+and+hepatocytes.+Nature+425%2C+968–973+(2003).) 2003 |
| Adult dermal fibroblasts | Induced pluripotent stem cells | Induced pluripotency [in vitro] | ~1% | [Tanabe K](http://www.ncbi.nlm.nih.gov/pubmed?term=Tanabe K%5BAuthor%5D&cauthor=true&cauthor_uid=23910081). et al. [*Proc. Natl. Acad. Sci.*](http://www.ncbi.nlm.nih.gov/pubmed?term=Kim J%2C et al.(2011) Direct reprogramming of mouse fibroblasts to neural progenitors. Proc Natl Acad Sci USA 108%3A7838–7843.) *U S A* 2013 |
| Adipose stromal cells | Induced pluripotent stem cells | Induced pluripotency [in vitro] | ~0.005% | [Narsinh KH](http://www.ncbi.nlm.nih.gov/pubmed?term=Narsinh KH%5BAuthor%5D&cauthor=true&cauthor_uid=23881547). et al. [*Nat. Protoc.*](http://www.ncbi.nlm.nih.gov/pubmed) 2011 |
| Adult dermal fibroblasts | Induced pluripotent stem cells | Induced pluripotency [in vitro] | ~0.01% | [Soldner F](http://www.ncbi.nlm.nih.gov/pubmed?term=Soldner F%5BAuthor%5D&cauthor=true&cauthor_uid=19269371). et al. [*Cell*](http://www.ncbi.nlm.nih.gov/pubmed/?term=Soldner+F%2C+et+al.+(2009)+Parkinson’s+disease+patient-derived+induced+pluripotent+stem+cells+free+of+viral+reprogramming+factors.+Cell+136%3A964–977.) 2009 |
| Fibroblasts | Induced pluripotent stem cells | Induced pluripotency [in vitro] | ~0.001% | [Kim D](http://www.ncbi.nlm.nih.gov/pubmed?term=Kim D%5BAuthor%5D&cauthor=true&cauthor_uid=23881547). et al. [*Cell Stem Cell*](http://www.ncbi.nlm.nih.gov/pubmed) 2009 |
| Dermal fibroblasts | Induced pluripotent stem cells | Induced pluripotency [in vitro] | ~0.01% | [Park IH](http://www.ncbi.nlm.nih.gov/pubmed?term=Park IH%5BAuthor%5D&cauthor=true&cauthor_uid=18157115). et al. [*Nature*](http://www.ncbi.nlm.nih.gov/pubmed/?term=Park%2C+I.H.%2C+Zhao%2C+R.%2C+West%2C+J.A.%2C+Yabuuchi%2C+A.%2C+Huo%2C+H.%2C+Ince%2C+T.A.%2C+Lerou%2C+P.H.%2C+Lensch%2C+M.W.%2C+and+Daley%2C+G.Q.+(2008).+Nature+451%2C+141–146.) 2008 |
| Embryonic fibroblasts | Induced pluripotent stem cells | Induced pluripotency [in vitro] | ~1% | Yusa K. et al. *Nat. Methods* 2009 |
| Fibroblasts | Induced pluripotent stem cells | Induced pluripotency [in vitro] | 0.1%< | Wernig M. et al. *Nature* 2007 |
| Adult fibroblasts | Live births  Live adults | Nuclear transfer | ~0.54%  ~0.36% | Kubota C. et al. [*Proc. Natl. Acad. Sci.*](http://www.ncbi.nlm.nih.gov/pubmed?term=Kim J%2C et al.(2011) Direct reprogramming of mouse fibroblasts to neural progenitors. Proc Natl Acad Sci USA 108%3A7838–7843.) *U S A* 2000 |
| Hematopoietic stem cells  Granulocytes | Cloned mice | Nuclear transfer | 0%  0.5% | Sung LY. et al. *Nat. Genet.* 2006 |
| Fetal ﬁbroblasts | Cloned term rats | Nuclear transfer | 1.7% | Zhou Q. et al. *Science* 2003 |
| Adult granulosa cells | Viable piglets | Double nuclear transfer | ~1.2% | [Polejaeva IA](http://www.ncbi.nlm.nih.gov/pubmed?term=Polejaeva IA%5BAuthor%5D&cauthor=true&cauthor_uid=10993078). et al. [*Nature*](http://www.ncbi.nlm.nih.gov/pubmed/?term=Polejaeva+IA%2C+Chen+SH%2C+Vaught+TD%2C+Page+RL%2C+Mullins+J%2C+Ball+S%2C+Dai+Y%2C+Boone+J%2C+Walker+S%2C+Ayares+DL%2C+Colman+A%2C+Campbell+KH.+2000.+Cloned+pigs+produced+by+nuclear+transfer+from+adult+somatic+cells.+Nature+407%3A86+–90.) 2000 |
| Germline stem cells | Pups born | Tetraploid complementation | 0% | [Ko K](http://www.ncbi.nlm.nih.gov/pubmed?term=Ko K%5BAuthor%5D&cauthor=true&cauthor_uid=19570517). et al. [*Cell Stem Cell*](http://www.ncbi.nlm.nih.gov/pubmed/?term=Ko+K%2C+Tapia+N%2C+Wu+G+et+al.+Induction+of+pluripotency+in+adult+unipotent+germline+stem+cells.+Cell+Stem+Cell+2009%3B5%3A87–96.) 2009 |
